# Effects of Lifestyle Factors on Cognition in Minority Population of Older Adults: A Review

**DOI:** 10.3389/fnut.2022.841070

**Published:** 2022-03-16

**Authors:** Jacob M. Eubank, Douglas J. Oberlin, Andrew Alto, Nadine R. Sahyoun, Elmira Asongwed, Lillie Monroe-Lord, Elgloria A. Harrison

**Affiliations:** ^1^Lehman College, City University of New York, New York, NY, United States; ^2^Department of Nutrition and Food Science, University of Maryland College Park, College Park, MD, United States; ^3^College of Agriculture, Urban Sustainability and Environmental Sciences, University of the District of Columbia, Washington, DC, United States

**Keywords:** cognition, brain health, dementia, minority populations, lifestyle factors

## Abstract

The onset of dementia and Alzheimer's disease (AD) is projected to expand over the next several decades in the United States as the population ages. However, the cognitive health burden is not equally distributed among the population, as Hispanics and African Americans are at higher risk of AD when compared with Non-Hispanic Whites. There is some evidence to indicate that cognitive decline may be associated with lifestyle factors and that interventions in these domains may prevent or delay this decline. These lifestyle factors include social engagement, physical activity, both aerobic and strength training, dietary intake, sleep and stress. This review summarizes, in general, what is known about the relationship between risk factors and cognition and, in particular what is known about this relationship in minority populations. The results show that the relationship between these risk factors and cognitive decline is stronger for some of the factors such as physical activity and dietary intake and weaker for the other factors depending on what is measured and in what populations. It does appear, however, that the studies in minority populations is limited and warrants more targeted research and interventions.

## Introduction

Through the lifespan, humans undergo various physiological and psychological changes. Changes in brain function and structure negatively affects cognitive and physical capabilities. The rate of decline is difficult to understand as individual differences extend across an entire lifetime. In addition to challenges based in individual variation, there are several different measures of cognitive function which may change with aging. Crystalized abilities tend to continue improving throughout the lifespan in a cumulative manner, while fluid abilities may be decreasing over the entire lifespan (1). Cognitive decline may begin as early as the age of 30 and 40 or may well extend into the 50s or 60s, depending on various measures and study designs (1–4). It is uncertain how much cognitive decline is unavoidable or able to be modified due to a lifetime of confounding factors (5–8). The question of cognitive decline is of primary concern as it can have an impact on every area of an older adult's life. Some cognitive areas of particular concern include attention, sustained attention, selective attention (9), and memory.

Aging and cognitive disfunction has been inextricably linked as a cause of health decline in older adults. This will lead to a greater public health burden as it is anticipated that by the year 2050 (10) there will be two billion older adults in the world. Several lifestyle factors have been linked to cognitive function/decline such as hypertension, obesity/diabetes, smoking, excessive alcohol consumption, social isolation, and inactivity (11, 12). While some of these risk factors cannot be directly modified, activities such as smoking or engaging in physical activity (PA) can. The Finnish Geriatric Intervention Study to Prevent Cognitive Impairment and Disability (FINGER) study provided evidence over a 2-year intervention that lifestyle factors can influence the onset of cognitive decline with many older adults suffering from Alzheimer's and other dementia diseases (11). The World-Wide FINGER (WW FINGER) study encouraged other researchers to conduct additional studies around the world to validate findings in more diverse populations of older adults (13). The U.S. Study to Protect Brain Health through Lifestyle Intervention to Reduce Risk (U.S. POINTER) is an offshoot of the WW FINGER Study with its goal of examining multidomain interventions among older adults in a racially balanced group in the United States (14).

The question of cognitive decline may also be an important public health issue to answer as the health burden of dementia and Alzheimer's disease (AD) is projected to expand over the next several decades as the United States population ages (15, 16). Additionally, the cognitive health burden is not equally distributed among the population, as Hispanics and African Americans have a higher rate of AD per total population when compared with Non-Hispanic Whites (15–17). These trends present a need for increased research to prevent and/or treat cognitive decline and dementia among these understudied but overly affected populations.

The purpose of our review is to determine available research on cognitive decline leading to Alzheimer's disease or dementia in large groups of African American and Hispanic American older adults. We chose African Americans and Hispanic Americans as our targeted population as our current work as researchers reflect heavily this population of older adults and because they are the largest minority groups in the US (18). Much of the current research shows that African Americans suffer a heavy burden from Alzheimer's disease compared to other groups and are less able to receive the proper care for this condition (19). Our main goal was to learn the state of research on African and Hispanic Americans that focused on social engagement, physical activity, diet, sleep, and stress as these relate to cognitive decline. While age is the biggest risk factor for cognitive decline, the phases of cognitive decline vary from asymptomatic preclinical phase, a symptomatic predementia phase (cognitive impairment, MCI), and a dementia phase (19). Alzheimer's Disease is a chronic disease that begins with abnormal proteins in the brain where cognitive impairment is not detected, followed by slight impairment (19). These are some examples of how the researchers measured cognitive decline in many of the studies reviewed: the Modified Mini Mental State Examination, Digit Span Forward Subtest and the Digit Symbol, and the Subtest of the Wechsler Adult Intelligence Scale.

Our review begins with an update of the literature on risk factors for cognitive decline such as social engagement, physical activity and exercise, dietary intake, sleep, and stress. Each section begins with the current state of research on each of these areas followed by examples of studies highlighting our targeted population. In this review, we acknowledge that an established relationship between chronic disease and cognition exists, and many studies have examined these in African American and Hispanic American older adults. We highlight exemplar studies that represent African and Hispanic American older adults Our study represents evidence-based research in human subjects only, included if the research study was conducted between 2005 to 2021, in English, and within the United States. There are occasions we cite seminal work of authors to address definitions used throughout our review. Our review reflects a narrative review and not a systematic review, therefore our researchers limited our search to mainly on open-source databases primarily Pub-Med and Google Scholar where the research studies were in open access journals. We confined our search to five key parameters with words combinations as noted in Table 1 below. Due to the modest nature of the search results in each category, studies focused on minority populations were limited. Selected studies were based on the following criteria: (1) sample included minority populations, (2) longitudinal design, and (3) positive outcomes such as improved memory, reduced depression, reduced rate of cognitive decline, and improved cognition.

**Table 1:** Key Parameters of Cognition.

**Key parameter**	**Search terms**
Social engagement	Sense of belonging, sense of community, social engagement, third-place, older adults, aging adults, African Americans
Physical activity, exercise, short-term memory	Mood, cognitive, exercise, aerobic, physical activity, older and older adults, resistance, short term memory, cognition, brain health, African American
Stress	Stress, cognitive decline, older adults, African Americans, and Hispanic Americans
Diet	Diet, cognitive decline, older adults, African Americans, and Hispanic Americans
Sleep	Sleep, cognitive decline, older adults, African Americans, and Hispanic Americans

## Social Engagement and Cognitive Decline

Social isolation defined as “objective and quantifiable reflection of reduced social network size and paucity of social contact” and loneliness defined as “reflecting the individual's experience dissatisfaction with the frequency and closeness of their social contacts or the discrepancy between the relationships they have and the relationships they would like to have.” This experienced dissatisfaction is associated with various physical ailments (i.e., increase in cardiovascular disease, elevated blood pressure, infectious illness, and mortality) (20), and psychological problems such as anxiety, stress, and depression which contribute to cognitive decline (21–23). We highlight in this section the importance of social engagement and activities, aging in place, neighborhood characteristics, and sites of significance, and key components of older adults aging in place.

### Studies With Minority Population

Social engagement improves well being and cognitive function in community dwelling older adults, however, previous studies have not specifically investigated the outcomes in Black, Hispanic, and Asian communities. In fact, there is a lack of research focused on these groups (24) conducted a study that explored social engagement and cognitive function in 617 older African American adults ages 57 to 97 years old. The researchers found that social engagement contributed to improved cognitive function. Pugh et al. further suggested research that investigates social activity and engagement interventions on cognition, size of social networks over time, and loneliness. Social engagement that builds social capital improves health and well being through reduction in stress and depression, which further reduces cognitive decline (25, 26).

### Aging in Place

“Third places” and “aging in place” have an important role in the lives of individuals who live in communities and neighborhoods and is associated with cognitive functioning. The “third place” (e.g., bookstore, coffeeshop, library, social club) refers to a location that acts as a social capacity for an individual, with the “first place” being the home, and the “second place” being the workplace (27). However, for recently retired and older adults, “third places” become the “second place” (28). “Third places” contribute to an individual's perceived sense of social cohesion (29, 30) particularly for those “aging in place” (older community dwelling adults) who find valuable intersections of social connection and interaction with others (31, 32) investigated the concept of aging in place in relation to the home, neighborhood, and community among 121 non-Hispanic White older adults 56–92 years old. They found that aging in place formed a sense of attachment to the home and the community for which the older individuals lived. The above studies identified the importance of “third places” and their role in providing a sense of place for older adults. However, none of the studies focus on the role of “third places” as a sense of place in reducing cognitive decline in older Blacks/African Americans and Hispanic Americans.

### Neighborhood Characteristics

Neighborhood characteristics can also have an impact on aging in place and sense of belonging, the perception that a person is an integral part of a social environment or system (33). A sense of belonging is a necessity in the human experience and is essential for psychological well being (34). Furthermore, sense of community belonging is defined as “the subjective perspective of personal integration into a neighborhood” (35). Interactions with neighbors, service individuals, and even strangers in the neighborhood provide a social network that promotes social relationships and overall well being (36).

Pauly et al. (37) explored the effects of neighborhood characteristics on social relationships and isolation in 97 older adults ages 50–85 years. In the sample of mostly white participants (75%), individuals with higher quality social relationships were less negatively impacted compared to those with lower quality social relationships; and those with higher quality social relationships were less socially isolated. Torres (38) investigated the impacts of gentrification on the ability of long-time residents in a New York City neighborhood to age in place. For the 47 men and women aged 60 years and older, findings indicated proximity, cost, design, and layout of these neighborhoods can influence how older adults age in place. Lee and Waite (39) explored the relationship between aging in place and cognition in a sample of 80% White-non-Hispanics, 10% Black, and 7% Hispanics. They found that certain qualities of the surrounding neighborhood influences cognition. Further, these researchers also found that perceived neighborhood danger, social ties, and social cohesion had a significant influence on cognition.

### Studies in Minority Populations in Sites of Significance

Gonyea et al. (35) explored the relationship between neighborhood characteristics and a sense of community belonging in 216 older adults with 50% of the sample identifying as Black and 45% of the sample identifying as Hispanics. They found that 80% felt safe in their neighborhoods during the day and 63% felt safe at night. Sixty percent felt a strong sense of community belonging, however, 26% experienced symptoms of depression. Unfortunately, this study did not examine how these neighborhood perceptions affected cognition among these individuals.

### Sites of Significance

Many older adults have a limited level of physical mobility that impacts the distance they can travel (36–38). “Sites of significance” (e.g., public parks); “Thresholds” (e.g., porches, backyards, balconies); and “transitory zones” (e.g., sidewalks, lobbies, subway platforms) provide access to social interactions for many older adults. Thus, proximity to sites of significance has a central role in providing a sense of belonging to older adults. Gardner (36) investigated how neighborhoods influenced aging and well being in six adults with an average age of 82.5 years. Interactions in sites of significance, thresholds, and transitory zones provided the participants a sense of purpose and positive outlook on life (36), however, all participants in the study identified as white. A sense of purpose is defined as “goals, intentions, and a sense of direction, all of which contribute to the feeling that life is meaningful” (40) and is positively associated with improved speed and memory, self-rated health, and a reduction in depression (41). Public spaces provide a sense of belonging with the community for older individuals who are aging in place. Additionally, there are many opportunities that promote interactions such as proximity and available social activities.

Sites of significance may be attractive to older adults due to the nature of the social activities that happen there. They provide a permanent and familiar place for activities such as a bridge club or playing sports or exercising (36, 38, 42–45) compared the contribution of individual vs. group social engagement among 3,413 older adults and found that group engagement positively impacted cognition compared to individual engagement. Brady et al. (42) investigated the impact of membership to a fitness program on social isolation and loneliness in a mostly White sample of 3,457 older individuals aged 65 years to 90 years of age. They found that membership to a fitness program drastically reduces social isolation and loneliness. Inoue et al. (44) explored the sense of belonging and well being among 50 participants aged 64 to 84 years through their engagement in spectator sports. Inoue et al. (44) found that frequent group attendance to sports games positively impacts sense of belonging and well being in older adults. Small et al. (46) found that a decrease in social activities and social engagement had detrimental effects on cognitive functioning in a sample of 952 older adults. The above studies suggest that social activities and social engagement are vital in the cognition of older adults, however, none of these studies focused on older Blacks/African Americans or Hispanic Americans.

### Studies in Minority Populations in Social Engagement/Activities

Ahn et al. (47) noted that more non-Hispanic White older adults engage in formally organized volunteerism compared to African Americans and Hispanic Americans. This study further showed 56% non-Hispanic whites, 51% African Americans, and 43% Hispanics engaged in volunteerism. It is interesting to note that Amano et al. (48) found that older Blacks/African Americans of lower economic status do not engage in these formal social activities as much as their White or Hispanic counterparts, and the researchers recommend further investigation of how racial and cultural identities influence engagement in formal social activities.

Older individuals living alone in residential neighborhoods are increasingly at risk of experiencing social isolation and loneliness (22, 23, 43, 49), which can have detrimental effects on cognitive health, mortality, stress, depression, and a general decline in health and well being (22, 23, 35, 36, 41, 43, 50). Older adults with functional limitations are less likely to have contact with friends and family. Similarly, older adults that are living in an independent setting are also less likely to have social contact with friends and family. The negative impacts of older adults living alone are further exacerbated for low-income people of color (51). A strong sense of belonging in the community provides many protective psychological factors against anxiety, stress, and depression while increasing one's overall health and well being (33, 40, 52). While neighborhood characteristics adds a concrete dimension to the health and wellness discussion, multiple studies have demonstrated the benefit of physical activity as a protective factor in improving cognitive decline.

## Physical Activities & Exercise and Cognitive Decline

A lack of PA increases the risk of several negative chronic health conditions, including cognitive decline and dementia (53–57). While PA only accounts for a population attributable fraction of 1.6–9.6% of dementia cases, several other dementia risk factors, such as hypertension, obesity, or diabetes, are themselves related to PA (12).

### Aerobic Training/Physical Activity Focus on Dementia

Among primarily non-Hispanic White participants, several cognitive parameters are improved with PA/exercise including memory, executive function, attention, and processing speed (58, 59). It is likely that the age when PA begins, overall fitness, and the total volume of PA all contribute to some aspects of these cognitive changes (60, 61). Despite these caveats, choosing to begin, or increase, PA at any age is likely to reduce risk of cognitive decline, in addition to other health benefits of PA (56, 62). A recent study of individuals ranging in age from 20 to 67 showed improved executive function following 6 months of aerobic exercise training, with greater improvements among the older individuals compared to the younger individuals (63). Individuals who are more fit and active early in life reduce their risk of early onset dementia and cognitive decline (12, 56, 64). However, otherwise healthy older adults were also able to improve their cognitive functioning by engaging in aerobic training over 2–6 months (62, 65).

### Aerobic Training/Physical Activity Focus on Memory

Aerobic exercise and fitness also relate to improvements in memory functions (61, 65–70). It has long been understood that aerobic exercise can delay the loss of memory, however recent studies have shown that as little as 12 weeks of higher intensity aerobic exercise may improve memory among older adults (68). Among healthy older adults, or older adults with mild cognitive impairment, 6 months of moderate intensity exercise was also sufficient to improve memory performance (65, 69).

### Aerobic Training/Physical Activity Focus Depression

In addition to cognition, PA is related to several mood parameters (71–74). Among healthy older adults, 10–12 weeks of physical activity was shown to reduce depression and improve vigor (71, 72). The effectiveness of physical activity to reduce depression has even been shown among community dwelling older adults, who are known to have high rates of depression (73, 74). Furthermore, moderate intensity exercise training over 12 weeks significantly lowered depression, anger, fatigue, and tension in older adults with pre-intervention depression (71). This is relevant, as depression itself is a potential contributor to cognitive decline and dementia (12). Hence, aerobic exercise and PA may be effective at improving mood outcomes as well as slowing the rate of cognitive decline.

### Studies in Minority Populations Using Aerobic Exercise or Physical Activity

Current research suggests that despite the health inequities among underrepresented populations, and associations between PA and cognitive decline, few studies have investigated the effects of PA and exercise on cognition among these marginalized groups. This may be due to a lower trust in medical research or fears of being mistreated among these communities (15). However, those studies that have investigated marginalized groups, show promising findings like studies of predominantly Non-Hispanic White populations. While most studies have focused on primarily non-Hispanic Whites some of the same benefits have been observed in the few studies that focused on older African Americans (75–77). Older African Americans display associations of executive function, learning and memory, and generalizing previous knowledge to novel problems with cardiorespiratory fitness levels (76). Cognitive decline was measured by scores on digit span and trail making tasks (executive function). There was also a correlation between cardiorespiratory fitness and BMI (when transformed to adjust for skew) and rey auditory verbal learning test (learning/memory). Beyond aerobic fitness, participation in PA is also shown to associate with cognitive parameters within older African-Americans (77). Among a cohort of 65-year-old African-Americans, even light intensity physical activity has been associated with higher cognitive processing (77). While some associations were observed for moderate to vigorous PA, too few participants were accumulating higher intensities of PA for associations to be assessed. The effects of exercise/PA extend beyond simple associations among these populations. Exercise interventions have also been used to elicit changes in cognitive function (75). A cohort of older African American individuals were divided into a cardio-dance or control group to participate in twice weekly exercise sessions. After 5 months of training, the dance group showed improvements in executive function, and reduced depression scores (75). This may have been more effective because the cohort had low fitness at the beginning of the study (75). Additionally, the group nature of the exercise intervention also allows effects of social engagement to influence the study outcomes. Regardless, these findings raise questions of why disparities exist in rates of cognitive decline between non-Hispanic White and African-American individuals when similar responses to PA are observed (78). We note, that while group exercise creates an environment that reduces social isolation, we focused here on the benefits of physical activity in improving cognitive function.

### Resistance Training

The current state of the research suggests less is understood regarding the temporal sequence of cognitive decline. There are some well known signs of the beginning of mild cognitive impairment (MCI) and cognitive decline, such as gait changes, working memory loss, verbal memory, and reduced visuospatial abilities (79). Some evidence suggests that cognitive damage is already done, and the focus should be on preventative measures to reduce or alleviate symptoms. Resistance training promotes several physiological changes associated with improved cognitive functions, risk reduction, and the severity of cognitive decline. Benefits include reduced neuroinflammation, reduced insulin resistance, and overall reduced adiposity (80). Interventions such as resistance training has been shown to be effective and economically beneficial; yet there are very few studies in African American and Hispanic American older adults.

Resistance training is often shown as a cost efficient and effective interventions for both preventing and reversing cognitive decline and cognitive impairment in older adults (81, 82), particularly in older adults with MCI, which is prevalent in about 20% of adults 60 years or older. Collectively, resistance training as a part of an intervention program is less costly, less invasive, and as beneficial when compared to pharmacological treatments that may have many side effects (83). The current state of research suggests that resistance training is vital in preventing cognitive decline in older adults, however, none of these studies focused on older African Americans or Hispanic Americans.

## Dietary Intake and Cognitive Decline

Lifestyle changes such as diet and nutrition have been shown in some research to slow the onset of Alzheimer's disease symptoms (84). Although the results are mixed, there is enough reliable evidence that suggest interventions that focus on diets have proven effective in non-Hispanic White older adults. There is however limited research in older adults who are African Americans or Hispanic Americans. Current research suggests a healthy diet may be a protective factor against cognitive decline (84, 85) while other research suggests that obesity is linked to age-related cognitive decline particularly in African Americans. A few research studies examined diet and cognition in minority populations.

### Studies in Minority Populations in Diet and Cognitive Decline

Sanchez-Flack et al. (86) reported that African Americans 65–74 have the highest rate of obesity in the United States. Building Research in diet and cognition (BRIDGE) Study was a randomized control trial of 185 African Americans using the Mediterranean Diet (MedDiet). This was a three-arm clinical trial intervention with MedDiet alone, MedDiet-A interventions, MedDiet-WL (weight loss calorie restrictions) (86). The results suggest that adherence to a MedDiet like pattern confers health benefits and reduces the risk of cognitive decline. Cognitive decline was measured by the Digit Span Forward Subtest and the Digit Symbol Subtest of the Wechsler Adult Intelligence Scale, and the California Verbal Learning Test (86). A prospective cohort study from the Health, Aging, and Body Composition study reported that black participants that had high MedDiet scores had lower rates of cognitive declines (87). Further, African Americans enrolled in the Chicago Health and Aging Project (CHAP) from 1993 to 2012 consumed the Mediterranean diet and showed a slower rate of cognitive decline (88). None of the studies noted in this review on diet and cognition focused on Hispanic Americans, further suggesting a gap in the research literature.

Research using the Chicago Health and Aging Project (CHAP) sampled a population of 2,280 Black and 1,510 White adults over 65 years with more than two (2) cognitive assessments, adherence to the Mediterranean dietary system with a minimum score of 55, and the Healthy Eating Index-2005 with a maximum score of 100. They found older adults who had higher scores had greater adherence to the Mediterranean diet. Further, these researchers noted that the higher the Mediterranean diet scores the slower the rate of cognitive decline (89). Additionally, Tsivgoulis et al. (90) sampled a cohort of 17,478 individuals enrolled in the REGARDS study who did not have a stroke, did not have cognitive impairment at baseline, and completed the Food Frequency Questionnaire in its entirety. These researchers reported no significant difference between race or place of residence; however, they noted a strong association of the incidence of cognitive impairment with those who had diabetes. The results showed the higher adherence to the Mediterranean diet the lower the incident of cognitive impairment in non-diabetic participants.

The Washington Heights-Inwood Columbia Aging Project (WHICAP) New York City, a cohort of 1,393 cognitively normal older adults and 275 older adults who developed mild cognitive impairment from a racially balanced group of Blacks, Hispanics, and non-Hispanic White older adults were followed over a period of 4 years. The results showed that participants who adhered to the Mediterranean diet were associated with lower Alzheimer's Disease (AD) risk and lower chances of developing AD in the future. Further, results showed that Hispanics better adhered to the Mediterranean diet than Blacks/African Americans and non-Hispanic Whites (91).

## Sleep and Cognitive Decline

Research shows that the lack of adequate sleep impairs cognitive functions, although cross-sectional studies have shown mixed results. Most clinical studies focused on short term sleep deprivation as this has been recognized in the United States as a public health crisis (92). Less attention has focused on long-term sleep loss and cognitive decline. Chen et al. (92) conducted a prospective study on 7,444 community-dwelling women 65 to 80 years old with self-reported sleep duration within the Women's Health Initiative Memory Study (WHIM). They found that women who slept <6 h or >8 h were at risk for cognitive decline. Cuellar et al. (93) noted that falling asleep during the day and staying awake are common occurrences in older adults and 69% do not report sleep disturbance to health care providers (p. 254).

Cohen-Zion et al. (94) noted cognition function decline in older adults with sleep disordered breathing. This study examined older adults 65 years and older using in-home interviews and sleep recordings. The results suggest that cognitive function decline is associated with greater daytime sleepiness. Sleep disordered breathing is defined as repeated nighttime arousals associated with apneic events resulting in sleep fragmentation, excessive daytime sleepiness and intermittent hypoxemia. The current state of research is rich with studies that focus on the non-Hispanic Whites with very limited research on African American and Hispanic American older adults.

Interesting work from Paech et al. (95) who noted differences in sleep duration between Blacks/African Americans and Whites by documenting the role of ancestry on sleep and cognitive performance. They found that African Americans compared to their White counterparts slept significantly less and cognitively performed worse on performance tests such as decreased reaction time, increased sleepiness, and mood. These researchers compared these cognitive changes to patterns noted in jet-lag or shift work. This study included participants between 31 and 43 years of age, which was less represented of our target population of older adults.

### Studies in Minority Populations in Sleep and Cognitive Decline

The REasons for Geographic and Racial Differences in Stroke (REGARDS), was a large representative sample of black and white middle-aged adults that studied the demographic characteristics to determine health differences and sleep disparities among minority groups. This study considered race, education, income, sex, and geographic residence on short and long sleep duration (96). The results of this study found that shorter sleep duration was greatest among older black adults and oddly enough, black men with higher income were found to sleep less than whites in regions of the country outside of the Southeast and Appalachia. Moreover, additional findings also suggest racial differences between blacks compared to whites in the 2010 Sleep America poll, blacks slept on average 38 mins less during the weekdays compared to whites (97). There is evidence that more multi-disciplinary research should be conducted on African Americans and Hispanic Americans and development of interventions needed to slow the rate of cognitive decline minimizing the onset of Alzheimer's and other dementia diseases. The Hispanic Community Health Study/Study of Latinos conducted by Agudelo et al. (98) was a cross-section community-based study in a large Hispanic Latino middle to older adults and found that longer sleep-onset latency was associated with global cognitive decline, verbal learning, memory, and word fluency. Sleep patterns were measured by using seven days of wrist actigraphy (a wearable sleep test that tracks movement).

## Stress and Cognitive Decline

Current evidence defines stress as environmental events or demands that exceed a person's ability to cope, which affects an individual's physical and psychological well being (99). Turner et al. (99) used a battery of 19 cognitive tests that were administered at baseline and at annual intervals for up to 9 years (mean follow-up = 4 years), from which composite measures of global cognitive function and five specific cognitive domains were derived. Chronic stress left unchecked has the ability to erode cognitive functioning with subsequent memory loss. More recently, the level of noise has been implicated as a health hazard and an environmental stressor in older adults. Weuve et al. (100) suggests that community noise exposure may influence the risk of Alzheimer's disease. The Chicago Health and Aging Project (CHAP) assessed 5227 participants and found that the higher level of residential noise was associated with worse cognitive performance. Most participants in the CHAP study were African Americans (63%), non-Hispanic White (36%), and Hispanic Whites and others (1.4%), which was one of the first of its kind study in the United States. Perceived discrimination is another area of research where psychological stress has detrimental effect on cognitive function. Most studies of psychosocial stress have been studied in Whites and less is known about other minority groups.

### Studies in Minority Populations in Stress and Cognitive Decline

Barnes et al. (101) studied a cohort of 407 older adult African Americans without dementia with a mean age of 73 years. They found that the higher level of perceived discrimination the worse cognitive performance in episodic memory and speed performance. The Wisconsin Registry for Alzheimer's Prevention (WRAP) study showed that the environmental and lived experiences differ between African Americans and Whites. This may lead to different stress-related life events that may impact health outcomes (102). WRAP study measured the level of performance on cognitive tests such as Speed & Flexibility and found that African Americans performed worse on these cognitive tests than whites. They saw a greater association between stressful life events and episodic memory; the greater the number of events the greater the level of cognitive decline (102). The WRAP study confirmed and supported findings of other studies that suggest that life courses and socio-environmental factors in African Americans influence cognitive decline in older adults (102). Some research has noted the perception of discrimination as stress inducing and has influenced psychological well being and cognitive functioning in older adults (103, 104).

The Minority Aging Research Study of 467 community-dwelling African Americans average age of 73, without dementia at baseline were followed for nine (9) years to determine the association between perceived stress and cognitive decline. The results showed the greater the perceived stress the greater the decline in global cognition, episodic memory, and visuospatial ability (99). These researchers found that older African Americans with “higher levels of perceived stress had faster rates of cognitive decline than those with lower levels of perceived stress” (99).

## Limitations/Future Research/Interventions

We acknowledge that our narrative review has limitations in that it was not an exhaustive search of the literature on studies that primarily focused on older adults who were African Americans and Hispanic Americans living independently in the United States. We found that many studies used different age categories for older adults which varied significantly; some studies included adults as young as 50 years. Our review purposely did not include international studies that addressed the same foci as our review, and we limited our review to articles published between 2005 and 2021 inclusively to provide the most relevant up-to-date research available. The composition of our research team has the expertise to develop interventions that are implementable in our three institutions in this Northeast region. We anticipate that interventions that focus on increasing social engagement by introducing module-based concepts like mindful meditation, virtual reality gaming programs, and community-based interactive social events that includes resistant strength trainings will improve the quality of life for older adults who are African Americans and Hispanic Americans living independently in the United States.

We also acknowledge that there are challenges to conducting this type of research on older aged, minority populations. Research on Alzheimer's disease and treatment mainly focused on non-Hispanic white samples and research on minority populations has been limited due to cultural and historical barriers, including recruitment and retention (19, 105). Providing clear intent of the research and consistent efforts to engage samples of minority populations increases transparency (19, 105, 106). Minority populations struggle with past immoral research behavior and face additional barriers including non-English speaking participants, transportation, and family responsibilities (19, 105, 106). Additionally, older aged individuals report a lack of motivation, energy, self-confidence, and feel they are too old to enroll in research studies (106).

## Conclusion

Our review on social engagement, physical activity, diet, sleep, and stress in African American and Hispanic American older adults provides only a glimpse into the current state of research for these key elements that demonstrates the importance of aging in place. Our review affirms the FINGER study in that not much has been written on minority populations and what has been written is limited in the types of interventions that have proven successful in minimizing cognitive decline. Given our interests and current work with minority populations, the current research review provides us a framework from which to develop interventions that are culturally sensitive and linguistically appropriate for African Americans and Hispanic Americans with the intended result to improve cognitive functioning. Research to understand the effects of social engagement, physical activity, diet, sleep, and stress as a comprehensive program on cognitive decline in African Americans and Hispanic Americans is lacking. Interventional research including educational programs focused on strategies to improve social engagement, physical activity, diet, quality sleep, and stress reduction in the aging adult population is needed, particularly in African American and Hispanic American older adults living alone.

## Author Contributions

JE and EH conceptualized the review topics and finalized the submitted version. All authors participated in the review process, collected data, wrote a section of the initial draft, revised the manuscript, and approved the submitted version.

## Conflict of Interest

The authors declare that the research was conducted in the absence of any commercial or financial relationships that could be construed as a potential conflict of interest.

## Publisher's Note

All claims expressed in this article are solely those of the authors and do not necessarily represent those of their affiliated organizations, or those of the publisher, the editors and the reviewers. Any product that may be evaluated in this article, or claim that may be made by its manufacturer, is not guaranteed or endorsed by the publisher.
