# Supplementary material for: Effects of Lifestyle Factors on Cognition in Minority Population of Older Adults: A Review
Source: Front Nutr. 2022 Mar 16;9:841070. doi: 10.3389/fnut.2022.841070 (PMC8966895; doi:10.3389/fnut.2022.841070)
Supplement: Supplementary file 1 [file Table_1.docx]

**Supplementary Table S1**

**Examples of selected studies in minority populations**

| **Author(s)** | **Participants** | **Research Design** | **Results** | **Reference** |
| --- | --- | --- | --- | --- |
| **Social Engagement and Cognitive Decline** | | | |  |
| Amano, T., Morrow-Howell, N., & Park, S. (2020). | 1,227 participants  54% non-Hispanic White  27% non-Hispanic Black  16% Hispanic  3% Other | Longitudinal study using data from the Health and Retirement Study | Three patterns of social engagement were identified among older adults with mild cognitive impairment: informal social engagement only, formal and informal social engagement, and low social engagement. | [48] |
| Gonyea, J. G., Curley, A., Melekis, K., & Lee, Y. (2018). | 216 participants  50% Black  45% Latino/a  5% non-Hispanic White or Asian American/Pacific Islander  mean age 67.4  73% female | Qualitative study using interview methodology | 80% felt “very safe” during the day and 63% felt “very safe” at night.  60% felt a strong sense of community belonging | [35] |
| Gonyea, J. G., Curley, A., Melekis, K., Levine, N., & Lee, Y. (2018). | 216 participants  50% Black  45% Latino  5% non-Hispanic White or Asian American/Pacific Islander  mean age 67.4  73% female | Qualitative study using interview methodology | 26% possessed depressive symptoms  Loneliness contributed to 49% of depressive symptoms | [21] |
| Pavela, G. (2015). | 9,447 participants  88% White  12% non-White  61% Female | Second Longitudinal Study on Aging | Those with a higher number of functional limitations is associated with less social contact with friends and family | [51] |
| Pugh, E., De Vito, A., Divers, R., Robinson, A., Weitzner, D.S., Calamia, M. (2020). | 617 participants  100% African American | Minority Aging Research Study (MARS) is a longitudinal epidemiological study. | Social activity and loneliness is associated with improved memory over time | [24] |
| **Physical Activity, Exercise, Short-term Memory and Cognitive Decline** | | | |  |
| Fausto, B. A., Azimipour, S., Charles, L., Yarborough, C., Grullon, K., Hokett, E., Duberstein, P. R., & Gluck, M. A. (2021) | 64 participants  100% African American  81% female  mean age 68.9 years. | Community-based research: a university-community partnership known as the Aging & Brain Health Alliance. Neuropsychologoical tests and questionnaires were performed before and after 5 month dance intervention. | Dance group reduced depression scores and improvements in attention (measured via Trail Making Test A) | [75] |
| Fausto, B. A., & Gluck, M. A. (2020). | 402 participants  100% African American  84% female  Mean age 69.7 years | Community-based research: a university-community partnership known as the Aging & Brain Health Alliance. Assessed cardiorespiratory fitness using a 6-minute walk test as well as several neuropsychological tests and questionnaires. Hierarchical regressions were performed to compare lifestyle attributes with cognitive performance. | Cardiorespiratory fitness correlated with scores on digit span and trail making tasks (executive function).  There was also a correlation between cardiorespiratory fitness and BMI (when transformed to adjust for skew) and rey auditory verbal learning test (learning/memory) | [76] |
| Mayeda, E. R., Glymour, M. M., Quesenberry, C. P., & Whitmer, R. A. (2016). | 274,283 participants  75% Caucasian  8% Latinx  8% Asian Americans  7% African American  2% American Indian / Alaska Native  0.2% Pacific Islanders  55% female  -Baseline mean age 73.4 | Population study | African American and American Indians / Alaska Natives had the highest rates of dementia followed by Latinx, Pacific Islanders, and Caucasians with intermediate rates of dementia, and Asian Americans with the lowest incidence of dementia. | [17] |
| Gothe, N. P. (2021) | 110 participants  100% African American  87% female  Mean age 64.8 years | Cross-sectional study. Older African American Adults completed several neuropsychological tests after wearing accelerometers for 7 days to monitor physical activity. | Greater light or moderate physical activity was associated with better performance on n-back tasks. Light physical activity was also associated with Eriksen Flanker task. | [77] |
| **Diet and Cognitive Decline** | | | |  |
| Agarwal, P., Dhana, K., Barnes, L.L., Holland, T.M., Zhang, Y., Evans, D.A., Morris, M.C. (2021). | Chicago Health Aging Project (CHAP)  5001 participants  63% African Americans  63% female  36% male  Mean age 74  Two cognitive assessments at follow-up 6.3 years | Large prospective population-based cohort study | African Americans enrolled in the Chicago Health and Aging Project (CHAP) from 1993 to 2012 consumed the Mediterranean diet and showed a slower rate of cognitive decline | [88] |
| Boumenna, T., Scott, T.M., Lee, J.S., Zhan, X., Kriebel, D., Tucker, K.L., Palacios, N. (2021). | At baseline 1502  At 2-years 1258  At 8-years 573 | Boston Puerto Rican Health Study (BPRHS), cross-sectional and longitudinal study | Adherence to the MIND Diet was associated with better cognition at baseline and over the 8-year follow-up period, but was not associated with 8-year cognition trajectory. | [107] |
| Koyama, A., Houston, D. K., Simonsick, E. M., Lee, J. S., Ayonayon, H. N., Shahar, D. R., Rosano, C., Satterfield, S., & Yaffe, K. (2015). | The Health, Aging, Body Composition Study  2326 older adults  70-79 years at baseline followed over 8 years  38% were Black  51% women | Prospective cohort study | The Health, Aging, Body Composition Study followed 2326 older adults 70-79 years at baseline over 8 years where 38% were Black and 51% women who adhered to the Mediterranean diet with results showing a reduction in cognitive decline compared to their White counterpart | [87] |
| Sanchez-Flack, J.C., Tussing-Humphreys, L., Lamar, M., Gantuzzi, G., Schiffer, L., Blumstein, L., McLeod, A., Dakers, R., Strahan, D., Restrepo, L., Hemphill, N.O.N., Siegel, L., Antonic, M., Fitzgibbon, M. (2021) | BRIDGE RCT 3 (ARM)  185 participants  MedDiet WL 75  MedDiet A 73  TDC 37    Age range 55-85  BMI 30.0-50.0 kg/m^2^ | Three arm randomized control trial | Participants were mostly African Americans greater than 70 years, mostly female who achieved modest weight lost with an improvement in cognition compared to isocaloric MedDiet A (weight stabilization) and TDC (typical diet control) | [86] |
| Scarmeas, N., Stern, Y., Mayeux, R., Manly, J. J., Schupf, N., & Luchsinger, J. A. (2009) | Washington-Heights-Inwood Columbia Aging Project (WHICAP)  1393 Cognitive normal older adults; 482 Mild Cognitive Impairment (MCI)    Cognitive normal  Black=474  Hispanic=473  White=434  Other=7  MCI  Black=144  Hispanic=214  White=124  Other=0 | Multi-ethnic community-based cohort study | The results showed that participants who adhered to the Mediterranean diet was associated with lower Alzheimer Disease (AD) risk and lower chances of developing AD in the future. Further, results showed that Hispanics adhered to the Mediterranean diet greater than Blacks and non-Hispanic Whites (Scarmeas, et al., p. 5). | [91] |
| Tangney, C. C., Kwasny, M. J., Li, H., Wilson, R. S., Evans, D. A., & Morris, M. C. (2011). | Chicago Health and Aging Project (CHAP)  2280 Black older adults  1510 White older adults  Mean age 75 | Ongoing longitudinal study | Results showed older adults who had higher scores had greater adherence to the Mediterranean diet. These researchers found that the higher the Mediterranean diet scores the slower the rate of cognitive decline | [89] |
| Tsivgoulis G, Judd S, Letter AJ, Alexandrov AV, Howard G, Nahab F, Unverzagt FW, Moy C, Howard VJ, Kissela B, Wadley VG. (2013) | Low adherence to MeD=9181  Mean age 63.8  Male=3801  Female=5380  Black=2703  White=6478  Stoke Belt region=5276  Other region=3905  High adherence to MeD=8297  Mean age 65.1  Male=3747  Female=4550  Black=2665  White=5632  Stoke Belt region=4556  Other region=3741 | Prospective population-based cohort study | Results showed no significant difference between race or place of residence; however, strong association of the incidence of cognitive impairment with those who had diabetes. | [90] |
| **Sleep and Cognitive Decline** | | | |  |
| Agudelo, C., Tarraf, W., Wu, B., Wallace, D. M., Patel, S. R., Redline, S., Kaur, S., Daviglus, M., Zee, P. C., Simonelli, G., Mossavar-Rahmani, Y., Sotres-Alvarez, D., Zeng, D., Gallo, L. C., González, H. M., & Ramos, A. R. (2021) | HCHS/SOL  1035 adults  45-64 years of age  Hispanic ethnicity    Dominican= 11.82  Central American= 6.57  Cuban=25.21  Mexican=32.52  Puerto Rican=19.87  South American=4.02 | Multi-center community-based cohort study | Results showed that longer sleep-onset latency was associated with global cognitive function decline in verbal learning, verbal memory, and word fluency. | [98] |
| Petrov ME, Long DL, Grandner MA, MacDonald LA, Cribbet MR, Robbins R, Cundiff JM, Molano JR, Hoffmann CM, Wang X, Howard G, Howard VJ. (2020) | REGARDS study  Black = 7,547  White=12,341  Mean age 63.8  Sleep duration(h), n (%)  <6 2259 (11.4)  6.0 _6.99 4468 (22.5)  7.0 _7.99 6079 (30.6)  8.0 _8.99 5689 (28.6)  9 1393 (7.0) | Longitudinal retrospective study | The results of this study found that shorter sleep duration was greatest among black older adults and oddly enough, black men with higher income was found to sleep less than whites in regions of the country outside of the Southeast and Appalachia | [96] |
| **Stress and Cognitive Decline** | | | |  |
| Turner, A. D., James, B. D., Capuano, A. W., Aggarwal, N. T., & Barnes, L. L. (2017). | The Minority Aging Research Study  467 community-dwelling African Americans average age of 73 without dementia at baseline and followed for nine (9) years | Minority Aging Research Study (MARS) is a longitudinal epidemiological study. | Results showed the greater the perceived stress the greater the decline in global cognition, episodic memory, and visuospatial ability (Turner et al, 2017). Older African Americans with “higher levels of perceived stress had faster rates of cognitive decline than those with lower levels of perceived stress” (Turner et al, 2017, p. 6). | [99] |
| Zuelsdorff, M., Okonkwo, O. C., Norton, D., Barnes, L. L., Graham, K. L., Clark, L. R., Wyman, M. F., Benton, S. F., Gee, A., Lambrou, N., Johnson, S. C., & Gleason, C. E. (2020). | WRAP Study  N=1241  African American=50  White=1191  WRAP visits completed  African American=3.4  White=4.5 | Longitudinal study | WRAP study measured the level of performance on cognitive tests such as Speed & Flexibility and found that African American performed worst on these cognitive tests than whites. They saw a greater association between stressful life events and episodic memory; the greater the number of events the greater the level of cognitive decline (Zuelsdorff, 2020, p. 9). The WRAP study confirmed and supported findings of other study that suggest that life courses and socio-environmental factors in African Americans influence cognitive decline in older adults | [102] |
